# Reemergence of Classical Swine Fever, Japan, 2018

**DOI:** 10.3201/eid2506.181578

**Published:** 2019-06

**Authors:** Alexander Postel, Tatsuya Nishi, Ken-ichiro Kameyama, Denise Meyer, Oliver Suckstorff, Katsuhiko Fukai, Paul Becher

**Affiliations:** University of Veterinary Medicine, Hannover, Germany (A. Postel, D. Meyer, O. Suckstorff, P. Becher);; National Agriculture and Food Research Organization, Tokyo, Japan (T. Nishi, K. Kameyama, K. Fukai)

**Keywords:** Classical swine fever, outbreak, classical swine fever virus, pestivirus, pig, wild boar, Asia, Japan, viruses

## Abstract

In September 2018, classical swine fever reemerged in Japan after 26 years, affecting domestic pigs and wild boars. The causative virus belongs to the 2.1 subgenotype, which caused repeated outbreaks in eastern and Southeast Asia. Intensive surveillance of swine and vaccination of wild boars will help control and eradicate this disease in Japan.

Classical swine fever (CSF) is one of the economically most devastating diseases worldwide and is notifiable to the World Organisation for Animal Health (OIE). The presence of CSF in a pig population results in severe restrictions on international trade of pigs and pork products. Many countries have implemented compulsory eradication programs and perform intensive surveillance. Most countries with industrialized pig production and high biosecurity standards have achieved the OIE status of being CSF free, including Japan in 2015 (*1*). Nevertheless, CSF is endemic to many countries that have a high number of backyard pigs. Because wild boars are as susceptible to CSF virus (CSFV) as domestic pigs, eradication of CSF in wild boars is of epidemiologic value (*2*).

CSFV, a positive-sense RNA virus (family *Flaviviridae*, genus *Pestivirus*) is divided into 3 major genotypes (*1*–*3*) and several subgenotypes (*3*,*4*). In Europe, the more recent outbreaks were caused by genotype 2.1 (Lithuania, 2009 and 2011) and genotype 2.3 (Latvia, 2013–2015) (*5*). In Asia, recent outbreaks were caused mainly by CSFV genotypes 1.1, 2.1, 2.2, and 2.3.

The spread of African swine fever (ASF) across China in 2018 has increased awareness of ASF and CSF in Southeast Asia. During August 16–September 3, 2018, at a pig farm in Gifu city, Gifu Prefecture, Japan, ≈20 fattening pigs died. A veterinarian recognized that the pigs were weakened and inappetent; no clinical signs were detected before August 20. Staff from the Gifu prefectural animal hygiene service center collected and sent samples from the following animals to the National Institute of Animal Health (Tokyo, Japan) to test for ASF and CSF viruses: 6 live pigs on August 24, 1 dead pig on September 3, and 11 live pigs and 1 dead pig on September 8. The CSFV genome was detected by reverse transcription PCR and confirmed by nucleotide sequencing. Control measures comprised culling of ≈600 pigs from the infected farm, movement restrictions, disinfection, epidemiologic investigations, clinical and laboratory investigations of 13 farms with epidemiologic links, and intensified surveillance. On September 13, a dead wild boar was found in the restriction zone of the initial outbreak and was CSFV positive. By March 7, 2019, a total of 68 dead and 153 live wild boars in Gifu and Aichi Prefectures had been found to be CSFV positive.

The last CSF outbreak in Japan (Kumamoto Prefecture) occurred in 1992; since 2006, vaccination against CSF has been banned. The absence of CSF in Japan for 26 years strongly suggests reintroduction of the virus from outside Japan. To support epidemiologic investigations, we performed molecular typing based on the partial 5′ untranslated region (UTR) (150 nt) and on the complete E2 gene (1,119 nt) by using the CSF sequence database and the integrated tool for phylogenetic analysis (*3*,*6*). Most similar sequences identified by database search (GenBank, https://www.ncbi.nlm.nih.gov/nuccore; BLAST, https://blast.ncbi.nlm.nih.gov/Blast.cgi) were included in the analysis together with 15 complete E2 encoding sequences (GenBank accession nos. MK026451–65) newly generated from isolates originating from Japan (10 sequences in 1951–1986), Thailand (4 sequences in 2001, 2011, and 2012), and Vietnam (1 sequence in 2010) (Figure). Phylogenetic analyses revealed that the 2018 isolate from Japan belongs to genotype 2.1; the E2 (Figure) and 5′-UTR sequences (Appendix) were most closely related to CSFV detected in China during 2011–2015 (98%–99% identity in E2 sequences; Figure) and China and Mongolia during 2014–2015 (98%–99% identity in partial 5′-UTR; Appendix). Subsequently, a complete genome sequence of the index isolate was determined (GenBank accession no. LC425854); the closest genetic relationship (98.9% identity) was with 2 recent isolates (GenBank accession nos. MG387217–8) from Beijing, China (*7*). Members of this phylogenetic clade reportedly form an emerging group of moderately virulent CSFV that is becoming more prevalent in China (*8*,*9*). Despite good availability of sequence data from China, much less information is available from other countries in the region. Therefore, similar viruses may be in other countries in eastern and Southeast Asia. Additional CSFV sequences from previous outbreaks in Japan, Thailand, and Vietnam were only distantly related to the sequence of the isolate from Japan. Partial E2 and 5′UTR sequences (GenBank accession nos. LC425434–5) obtained from the first positive wild boar (index case) revealed 100% identity to the index isolate.

**Figure:**
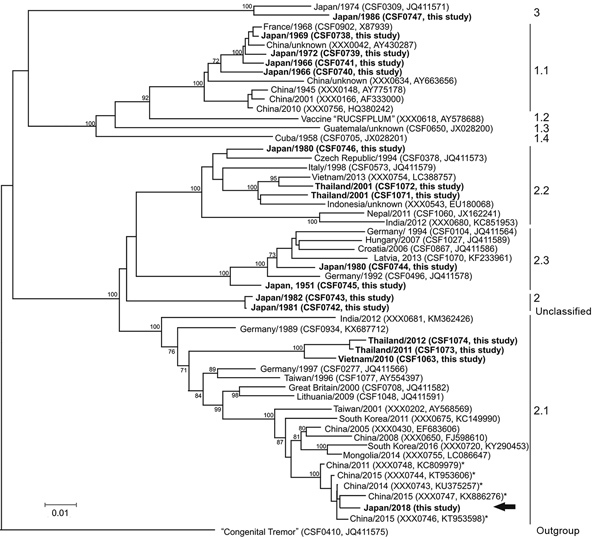
Phylogenetic tree displaying the genetic relatedness of the classical swine fever virus (CSFV) isolate obtained from the 2018 classical swine fever (CSF) outbreak in Japan to other CSFV isolates. Phylogenetic analyses were performed by using the neighbor-joining method with complete E2 (1,119 nt) sequences, including 1,000 iterations for bootstrap analysis, and were generated by the genetic typing module of the CSF database at the European Union and OIE Reference Laboratory for Classical Swine Fever (*6*). Only bootstrap values >70% are indicated. For each isolate, country and year are given, along with catalog number of the CSF database and the GenBank accession number in parentheses. The arrow indicates the sequence of the virus isolate obtained from a domestic pig during the 2018 CSF outbreak in Japan (Japan/2018) (GenBank accession no. LC425432). Boldface indicates additional newly generated sequences (GenBank accession nos. MK026451–65). Two ancient sequences from Japan (CSF0742, CSF0743) were identified as belonging to genotype 2 with no clear affiliation to any of the 3 established subgenotypes 2.1, 2.2, and 2.3 (indicated as genotype 2, unclassified). Asterisks indicate 5 E2 sequences belonging to a group of sequences that are most closely related to the E2 sequence of the CSFV isolate Japan/2018. Scale bar indicates nucleotide substitutions per site.

Japan is among the top 10 pork-producing countries worldwide; in 2017, an estimated 16.3 million pigs were slaughtered in Japan. Presence of CSFV in wild boars remains a serious threat for domestic pigs. By February 2019, the virus had further spread from Gifu Prefecture into other prefectures in Japan, emphasizing the need for defined strategies to control the outbreak, including vaccination of wild boar, in addition to the standard policy of culling. Moreover, intensive surveillance is needed to monitor the situation carefully and will contribute to the control and eradication of CSF in Japan.

Appendix Genetic relatedness of classical swine fever virus isolates, according to partial 5′ untranslated region sequences.
