# Supplementary material for: Reemergence of Classical Swine Fever, Japan, 2018
Source: Emerg Infect Dis. 2019 Jun;25(6):1228–31. doi: 10.3201/eid2506.181578 (PMC6537743; doi:10.3201/eid2506.181578)
Supplement: Appendix — Genetic relatedness of classical swine fever virus isolates, according to partial 5′ untranslated region sequences. [file 18-1578-Techapp-s1.pdf]

Article DOI: https://doi.org/10.3201/eid2506.181578

Reemergence of Classical Swine Fever, Japan, 2018

Appendix

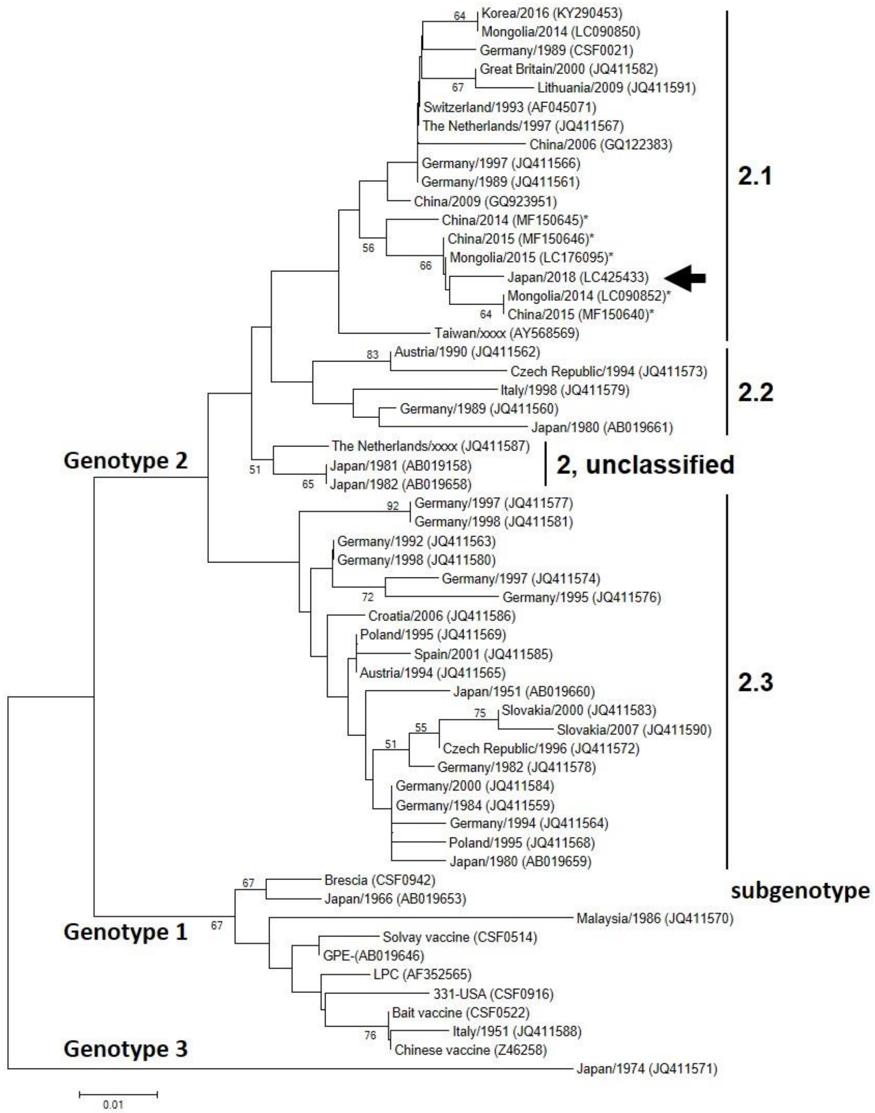

Appendix Figure. Phylogenetic tree based on partial 5'UTR sequences (150 nt). Analysis was performed by the neighbor-joining method including 1,000 iterations. Only bootstrap values ≥50% are indicated. For each isolate, country and year are given (GenBank accession number in paranthesis). The sequence of the virus isolate obtained from a domestic pig during the outbreak of CSF in Japan in 2018 (Japan/2018) is indicated by an arrow (GenBank LC425433). Two ancient sequences from Japan

(CSF0742, CSF0743) together with an ancient sequence from The Netherlands (JQ411587) were identified to belong to genotype 2, without clear affiliation to any of the three established subgenotypes 2.1, 2.2 and 2.3 (indicated as genotype 2, unclassified). Five sequences belonging to a group of sequences that are most closely related to the partial 5'UTR sequence of the CSFV isolate Japan/2018 (BLAST search, GenBank) are indicated by asterisks. In addition to three sequences from China, these comprise two sequences from Mongolia (Mongolia/2014 (LC090852) and Mongolia/2015 (LC176095) for which complete E2 encoding sequences were not available.
